# Influence of Layer Thickness on the Detection of Plastic Films Using Optical Photothermal Infrared Spectroscopy (O-PTIR)

**DOI:** 10.1021/acsmeasuresciau.5c00149

**Published:** 2026-01-22

**Authors:** Jan Fridtjof Häusler, Florian Bittner, Madina Shamsuyeva

**Affiliations:** IKK − Institute of Plastics and Circular Economy of the Leibniz University Hannover, An der Universität 2, 30823 Garbsen, Germany

**Keywords:** O-PTIR, IR spectroscopy, polymers, film, analysis

## Abstract

This study investigates how varying film thickness affects the qualitative identifiability of plastics using optical photothermal infrared spectroscopy (O-PTIR) on examples of common plastics such as polyamide 6 (PA6) and polyethylene terephthalate (PET). The main methodology consists of applying a thin layer of PA6 and PET separately to each substrate, namely, PET to polyethylene (PE) and PA6 to polypropylene (PP), and reducing the thickness of the coatings until the O-PTIR signal of the film is no longer detectable. As expected, the characteristic O-PTIR signal for PA and PET decreased with a decreasing film thickness. However, the results show that the O-PTIR detection limit for the plastics could not be reached, as the characteristic peaks of the substrate plastics are still clearly visible at a layer thickness of approximately 0.18 μm for PET and approximately 0.29 μm for PA6. These are the minimum stable film thicknesses that could be achieved since the selected film production process (drop deposition process) does not allow for thinner layers. As this work is a feasibility study, further factors influencing the O-PTIR measurement of plastic films should be investigated in a subsequent work.

## Graphical abstract

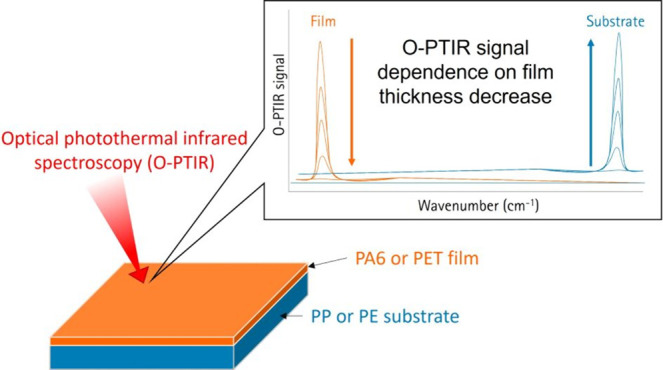

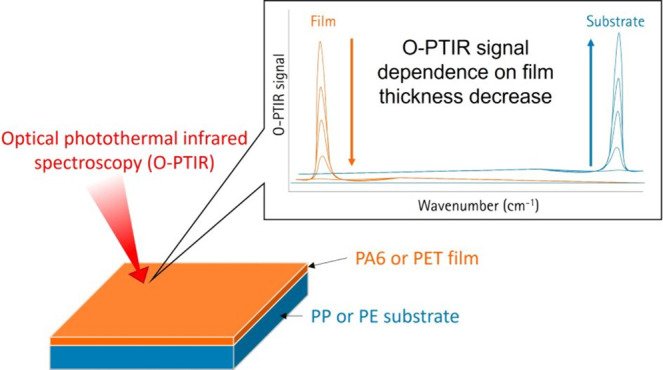

## 1 Introduction

The demand for fast and reliable methods for analyzing the chemical composition in the plastics industry is increasing as the composition of products becomes more complex and the diversity of materials used in plastics products increases. This includes combinations of different plastics, the use of various additives, coatings, etc. In particular, this diversity poses a major challenge for plastics recycling. It is therefore important to develop sensitive analytical methods to precisely and accurately determine contaminants and foreign substances and, on this basis, to develop more effective and efficient sorting, separation, and cleaning as well as recycling processes. In this context, the use of the O-PTIR method is a promising approach.

O-PTIR is a technology that forms the basis for the mIRage device. O-PTIR technology overcomes the IR diffraction limit that exists in conventional IR microscopy techniques such as Fourier transform infrared spectroscopy (FT-IR). In the O-PTIR technique, the specimen is irradiated with a pulsed, tunable quantum cascade laser (QCL) in the mid-IR range.^1,2^ The infrared absorption is measured indirectly with a visible laser beam. When the QCL laser is set to a wavelength that excites the molecular vibrations in the specimen, absorption occurs, resulting in photothermal effects, such as expansion of the specimen surface. The probe laser, which is focused on a point in the submicrometer range, detects this photothermal reaction by modulating the scattered light. In about two seconds, the IR laser can be moved across the entire range to generate an IR spectrum. The general principle of this measurement technology on various materials has already been described in the literature.^1,2^ Individual studies have already reported on the use of O-PTIR for the analysis of a thin layer poly­(methyl methacrylate) (PMMA) or 15.7 μm diameter PMMA microspheres,^2^ microplastics,^3–6^ paint layers,^7^ etc. However, to date, no systematic study has been conducted to investigate the penetration depth of the O-PTIR in plastics.

The aim of this study is therefore to investigate the minimum thickness for detection using two common plastics, PA6 and PET, which are applied separately as a coating, i.e., film to two different substrates, namely PP and PE. These plastics are selected for two reasons: (1) the characteristic O-PTIR peaks of PA6 and PET do not overlap with the characteristic peaks of PP and PE, (2) these are some of the most commonly used plastics with large market shares.^8^ They therefore provide a solid basis for further developing this analytical approach from a scientific perspective and prompt transfer into practice.

## 2 Experimental Section

In the first step, the PA6 and PET plastic granulates are dissolved separately in a solvent, hexafluoroisopropanol (HFIP), to produce solutions with varied concentrations. The optimal concentration range and film deposition method are determined in preliminary practical tests, as this approach is not described in the literature for PA6 and PET, and the approaches used in other studies conducted on plastics, such as PMMA or using other film deposition methods, such as spin coating, could not be directly applied in this study.^9^ Films of varied thicknesses are then produced from the individual solutions by dropping them onto the PE and PP substrates and evaporating the solvent at room temperature. The film thickness is mainly controlled by varying the concentration of the plastics in the solution. The manufactured film is characterized using digital microscopy.

It is important to note that the literature already contains extensive and detailed descriptions of the various factors that influence the process of polymer film formation and the morphology of the resulting films at different levels of detail, such as the effect of type and nature of solvents and solutes, substrates, and conditions such as temperature, humidity, etc. In addition, polymer concentration, evaporation rate and volatility of the solvent, viscosity of the solution, diffusivity of the solute, interactions between polymer–solvent and polymer–polymer and solvent–solvent molecules, molecular weight of the polymers, surface energy and topography of the substrate surface, etc.^9–13^ Since consideration of these aspects would go beyond the scope of this study, they are not examined in detail in this feasibility study, but care is taken to ensure that all environmental and process parameters remained unchanged during the experiment, except for the varied concentration. The manufactured plastic films are measured nondestructively using O-PTIR. The measured specimens are then subjected to the layer thickness measurement using scanning electron microscopy (SEM). Finally, by correlating the characteristic O-PTIR peaks with the varying PA6 and PET film thicknesses, the influence of film thickness on the detectability of plastics by using an O-PTIR is determined.

The following approach was already successfully used in the literature for the FTIR analysis of other films with varied thickness such as, for example, SiO_*x*_H_*y*_ on PP substrate,^14^ tetraglyme coatings on ultrahigh-molecular-weight PE substrate,^15^ and polystyrene (PS) on PET.^16^ Consequently, this study is based on the assumption that this approach also works for the O-PTIR microscopy. Although, unlike the FTIR measurements described in the above-mentioned literature, O-PTIR works in a reflection mode.

## 3 Specimen Preparation

First, the PE and PP parts with dimensions of 80 mm × 90 mm × 3 mm (length × width × height) are manufactured using the injection molding machine Allrounder 470A (Arburg, Germany). Afterward, with the help of a water jet cutting machine, Härtel Compact Basic (Härtel Laser + Wasser GmbH & Co. KG, Germany), the substrates with dimensions of 20 mm × 20 mm × 3 mm (length × width × height) are cut from the parts. Four substrates are cut from each part and cleaned with ethanol before the film manufacturing.

PA6 and PET granulates are dissolved in HFIP using the Agilent SP260VS heating and shaking unit (Agilent Technologies, USA) at a temperature of 24 °C. The dissolution of PA6 in HFIP takes approximately 24 h, and that of PET is approximately 72 h. This is in line with expectations because PA6 is more soluble in HFIP, particularly due to its strong interactions, e.g., via hydrogen bonds. Although PET dissolves in HFIP, ester groups, which are only hydrogen bond acceptors, result in a weaker interaction with HFIP. Even though there are no direct studies comparing the solubility of PA6 and PET under the same conditions, there are some studies that indirectly show the faster solubility of PA6.^17–19^

A drop deposition method is used to manufacture the polymer films. In the scope of preliminary tests, the possibility of using a spin coater is tested, which is a technique that is more commonly used for manufacturing polymer films. However, due to the poor compatibility between the nonpolar substrates PE and PP and the polar PA6 and PET, this technique proved to be unsuitable for this study. The dissolved plastics PA6 and PET are applied to the substrates in a volume of 7 μL using a single-channel microliter pipet Eppendorf Research plus (Eppendorf SE, Germany) at an angle of approximately 5°. The distance between the pipet and the substrate during application is the same for all test specimens and is approximately 1 cm. Films are prepared from the solutions with a plastic concentration of 1.0, 0.5, 0.25, 0.125, and 0.0625 wt % and named according to the nomenclature “[plastic content] [plastic type]/[substrate type]”. For example, “0.25_PET/PE” means that a PET solution with a content of 0.25 wt % is used to prepare a film on a PE substrate.

The production of polymer films from further diluted solutions, i.e., less than 0.0625 wt % of the dissolved plastics, was not possible using the drop separation method, as the adsorbed polymers did not form a homogeneous layer after the solvent had evaporated but rather discrete polymer particles or island-like domains. There may be several reasons for this behavior, some of which may also occur simultaneously. For example, the polymer volume may be insufficient to form a continuous film, and when the solvent evaporates, the polymer chains aggregate into localized domains rather than a uniform network. Similarly, capillary flow may carry away the polymer or leave behind island structures. At low concentrations, there may be too little polymer to distribute evenly. Furthermore, due to the incompatibility of surface energy between the plastic film and the substrate, dewatering may occur if the interfacial tension between the polymer and the substrate is unfavorable. As a result, the liquid does not spread evenly but retreats during solvent evaporation and breaks down into droplets.^20^ Similarly, phase separation may occur before the film dries, resulting in local areas with a high polymer content and others with a high solvent content, and when the solvent-rich areas evaporate, islands of polymer remain.^21^ Although practical identification of the reasons is not considered, a plastic concentration of 0.0625 wt % is ultimately selected for this study as the lowest concentration for film manufacture.

For the O-PTIR measurement of pure PA6 and PET (i.e., PA6 reference and PET reference specimens without PE and PP substrates), KBr windows are used. The reference films are produced on the KBr windows from the dissolved polymers with the highest concentration of 1 wt % under the same conditions as the tested films.

## 4 Instrumentation

### 4.1 Digital Microscopy

Selected specimens are subjected to digital microscopy measurement to ensure the quality of the manufactured plastic films. The films are examined using a Leica DVM6 A 3D digital microscope (Leica Microsystems, Germany). A PlanAPO FOV 43.75 lens, providing dark-field illumination, with a maximum resolution of 415 lp/mm, is used.

### 4.2 Optical Photothermal Infrared Spectroscopy

The O-PTIR microscope mirage from Photothermal Spectroscopy Corp. (USA) is used for the O-PTIR measurements. The O-PTIR software (version 4.3.7478) from the same manufacturer is used for data acquisition and processing. O-PTIR image excites the molecular vibrations of the specimen. In this study, the IR spectra are displayed at a wavenumber of 781 to 1801 cm^–1^. The measurement conditions are represented in Table 1.

**Table 1:** Parameters Used for the O-PTIR Measurement

**parameter**	PP substrate	PE substrate
IR power in % (∼10 mW)	10	10
probe power in %	10	10
detector gain in %	20	20
wavenumber sweep speed	1000 cm^–1^/s	1000 cm^–1^/s

Three film specimens are produced for each solution, and each film is measured three times. The three measuring points on the films are the same for all specimens. The O-PTIR spectra considered in the study therefore show the average of a total of nine recorded spectra, which is normalized to a maximum of 1. This means that the highest peak is set at an intensity of 1. The aim here is to make the data from individual spectra as comparable as possible. The comparison of all raw spectra is presented in the Supporting Information. A photographic record of the measurement setup and a graphical representation of the measured specimen can be seen in Figure 1.

**Figure 1:**
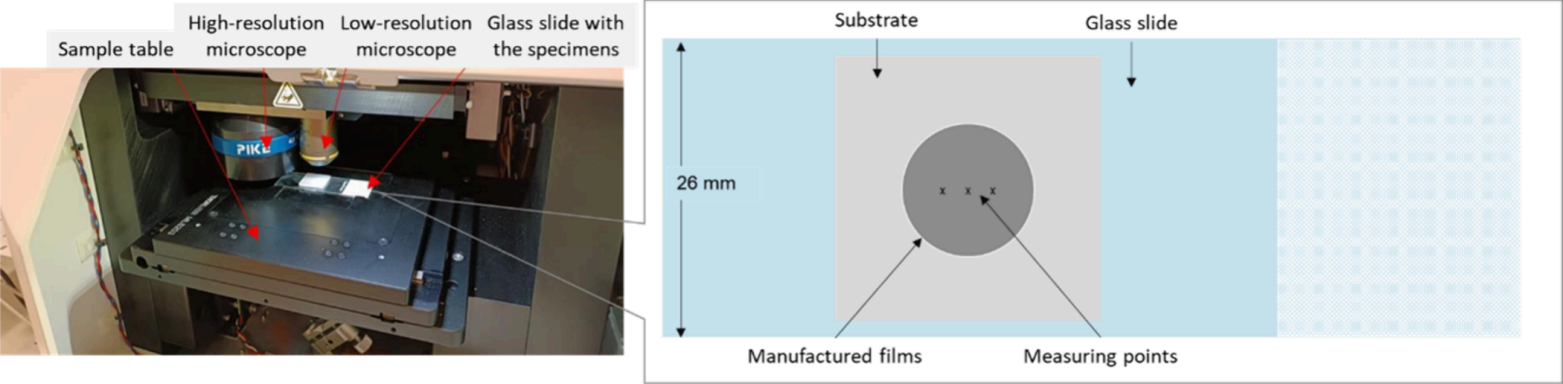
mIRage specimen table with specimen.

### 4.3 Scanning Electron Microscopy

To examine the selected specimens using SEM, we first embedded them in epoxy resin. The specimens are put in an embedding mold measuring 23 mm × 23 mm × 8 mm (length x width x height) (Plano, Germany) and fixed to the bottom of the mold with an adhesive, with the film coating facing upward. The mold is then filled with epoxy resin and left to cure at room temperature for 48 h. The precision cutting device Q-ATM QCUT 150 M (ATM QNESS GmbH, Germany) is used to cut the embedded specimen in the middle along the three O-PTIR measurement points (Figure 1, right). To make the specimens conductive for SEM examinations, a JEOL JFC-1300 sputter coater (JEOL Ltd./, Japan) is used to coat the specimens with a gold layer with a thickness of approximately 15 nm. Specimens are analyzed using a SEM JEOL JSM-IT510LA (JEOL Ltd., Japan) to measure the thickness of the manufactured polymer films. An accelerating voltage of 10 kV and a secondary electron detector are used to acquire the SEM images.

### 4.4 Materials

The high-density polyethylene (HDPE) under the brand name JUZEX 7303 used for the study is purchased from MSH Polymers (Germany). The PP with a trademark Moplen EP448T is supplied from Lyondell-Basell Industries (Germany). PA6 with a density of 1.14 g/cm^3^ under the brand name TEREZ B 305 is purchased from TER HELL Plastic GmbH (Germany). PET under the brand name Lighter PET C93 with a density of 1.34 g/cm^3^ is purchased from Equipolymers (Germany). HFIP with a purity suitable for analytical purposes is used as a solvent for the manufacture of PA6 and PET films. The casting resin used for the embedding of the specimens is a mixture of Araldit G2 epoxy resin and Aradur H2 hardener (Carl Roth GmbH + CO. KG, Germany) in a ratio of 1:10. For the manufacture of PET and PA reference specimens, a potassium bromide window from Bruker Optics GmbH & Co. KG is used.

The material criteria for selecting the polymers for this study are based on the fact that the characteristic peaks of the substrates and coatings do not overlap.

## 5 Results and Discussion

The reference O-PTIR spectra of the PE, PP, PA6, and PET used in this study are shown in Figure 2. The peaks marked with an arrow at wavenumbers of 1473 cm^–1^ for PE, 1377 cm^–1^ for PP, 1641 cm^–1^ for PA6, and 1721 cm^–1^ for PET are the characteristic peaks considered in this study. Overall, the O-PTIR spectra correspond very well with the spectra reported for these plastics in the literature.^22^ Minor deviations in the wavenumbers are due to differences in the settings of the measurement parameters and different additive packages in the individual plastics.

**Figure 2:**
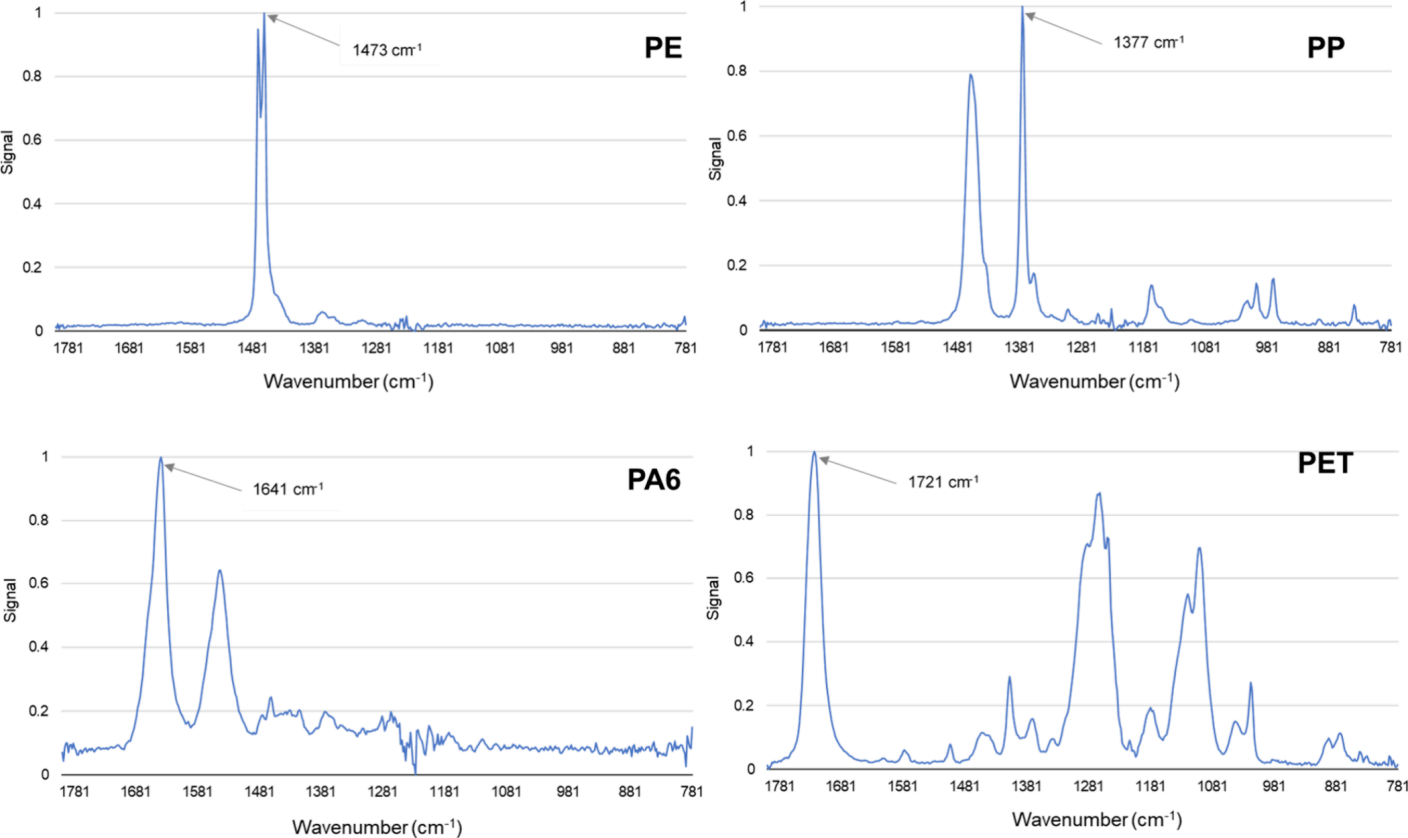
O-PTIR spectra of the plastics used in the study.

Figure 3 represents exemplary digital microscope images of selected film specimens and specifies the area of the films, in case it is distinguishable from the substrate. It is also observed that although all films are produced from the same volume of dissolved polymer, there are some differences between PET and PA6. As the PET concentration is reduced to 0.25 wt %, a change in the morphology of the film edges is observed in both cases: at higher concentrations of 1 wt %, the films are almost round with clearly recognizable film borders, while at 0.25 wt %, uneven film borders occur, leading to a so-called “coffee ring effect”.^23^ This effect arises because of contact line motion (spreading, evaporation, etc.) and is observed in the dried droplets of various dissolved materials, including polymers. The patterns are influenced not only by the type of polymer but also by their molecular weights and concentrations and can be controlled by using special additives.^24^ In addition, the lower concentrations lead to larger film areas both for PA6 and PET, but the increase is higher for PET. This observation can also be caused by various reasons, which may also occur simultaneously. For example, since the concentration of the polymer in the solvent varies in this study, the viscosity of the solutions also decreases with decreasing concentration, resulting in better spreading and a larger film area. Another effect that may explain the different spreading behavior of PET and PA6 and has already been described in the literature is the entanglement of the polymer chains. In particular, it has already been shown that PET in HFIP exhibits less chain entanglement or steric hindrance in solution, which allows for easier flow and spreading.^19^ These effects can also be influenced by molecular weight, additives in the plastic, and interfacial interactions, etc. Since determining these factors would detract from the focus of the study, the study concentrates mainly on the resulting film thickness and not on the factors that led to this result. At the lowest concentrations examined (0.625 wt %), no film layer could be detected in either PA6 or PET using digital microscopy, although when the solution drop is applied, it is possible to see that it spread further apart than at the higher concentrations. The film is probably too thin for a digital microscope, and the edges of the film show a more pronounced coffee ring effect, which further complicates the analysis. However, these samples are still examined using the O-PTIR.

**Figure 3:**
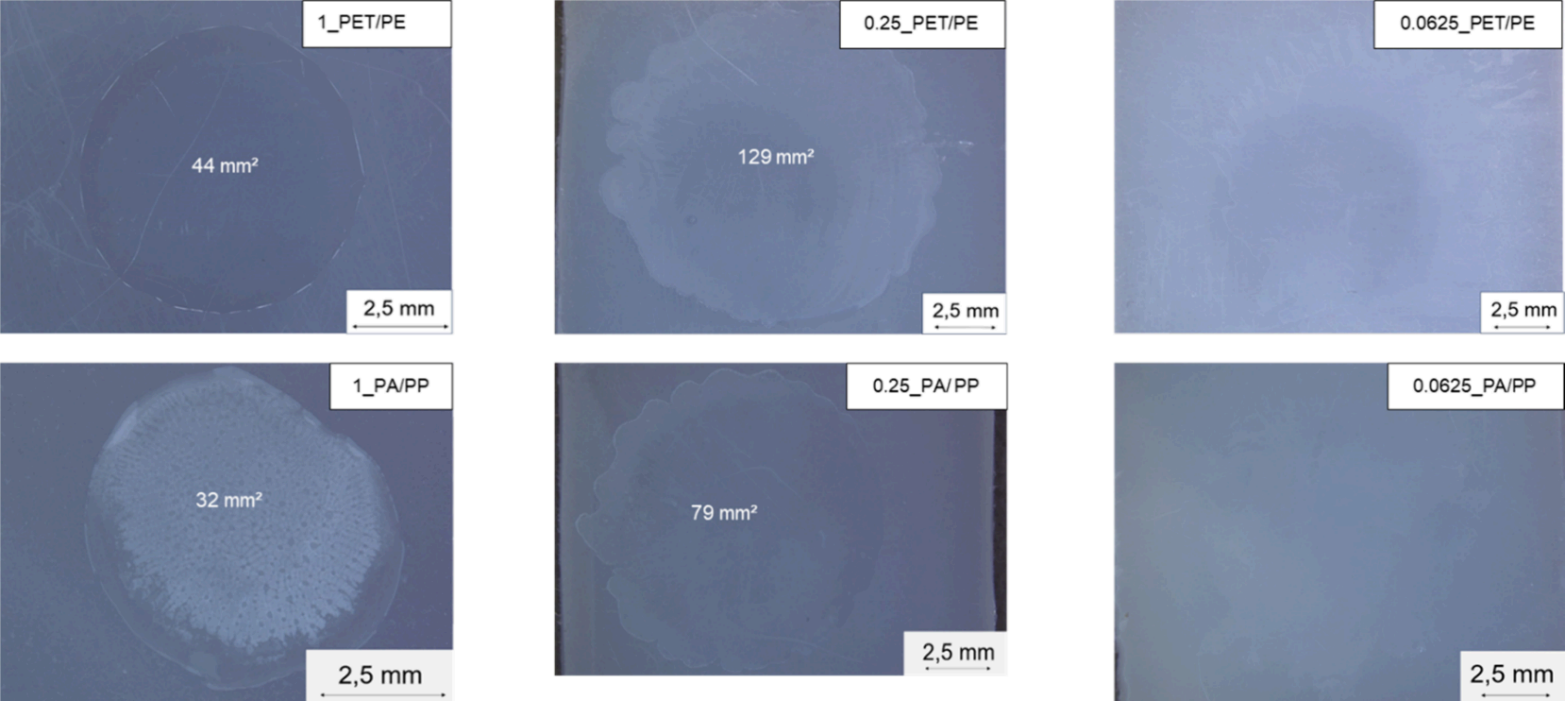
Digital microscopic images of selected PA and PET films on the substrates.

The measured film thicknesses for PET on the PE substrate and PA6 on the PP substrate are shown in Table 2. The SEM images are represented in the Supporting Information.

**Table 2:** Thickness of the Manufactured PET and PA Films

PET on a PE substrate	film thickness (μm)
1_PET/PE	14.35 ± 0.3
0.5_PET/PE	2.98 ± 0.8
0.25_PET/PE	0.91 ± 0.4
0.125_PET/PE	0.38 ± 0.2
0.0625_PET/PE	0.18 ± 0.1
	
PA on a PP substrate	film thickness (μm)
1_PA/PP	4.04 ± 0.1
0.5_PA/PP	1.51 ± 0.5
0.25_PA/PP	0.36 ± 0.1
0.125_PA/PP	0.29 ± 0.1
0.0625_PA/PP	the thickness could not be measured

Both PET and PA6 show an exponential decrease in the film thickness as a function of the polymer concentration, Figure 4. The behavior is in line with the results described extensively in the literature on film morphology studies using spin-coating film deposition.^10,12^ Specifically, the layer thickness of polymers increases with increasing concentration, but since concentration does not affect viscosity linearly, it is not a simple proportionality. The exact scaling depends on the polymer–solvent system, the spin speed, the evaporation rate, and whether entanglement and concentration ranges are exceeded.

**Figure 4:**
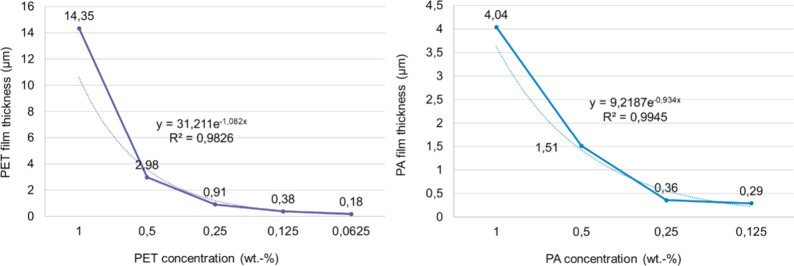
PET (left) and PA6 (right) film thickness as a function of the polymer concentration in HFIP.

The results of the O-PTIR measurements are shown in Figure 5. The characteristic peaks are identified with peaks for the individual combinations of polymer film and substrate. In general, these results show that the thinner the polymer films are, the lower the intensity of the characteristic peaks of PA6 and PET, and the higher the intensity of the characteristic peaks of the substrates PP and PE. At the same time, even at the lowest concentrations, a signal from PA6 and PET can still be observed, which is particularly evident in PET/PE. In other combinations, the change in substrates’ peak intensity is less smooth and tends to jump from higher concentrations (or film thicknesses) to lower concentrations (or film thicknesses). Similarly, the peak intensities for PA6 (1641 cm^–1^) and PET (1721 cm^–1^) decrease with decreasing plastic concentration in the solutions, but this decrease is proportional to neither the plastic concentration in the solution nor the film area in Figure 3. Consequently, the next step involves a direct measurement of the film thicknesses using SEM and correlating them with the O-PTIR results.

**Figure 5:**
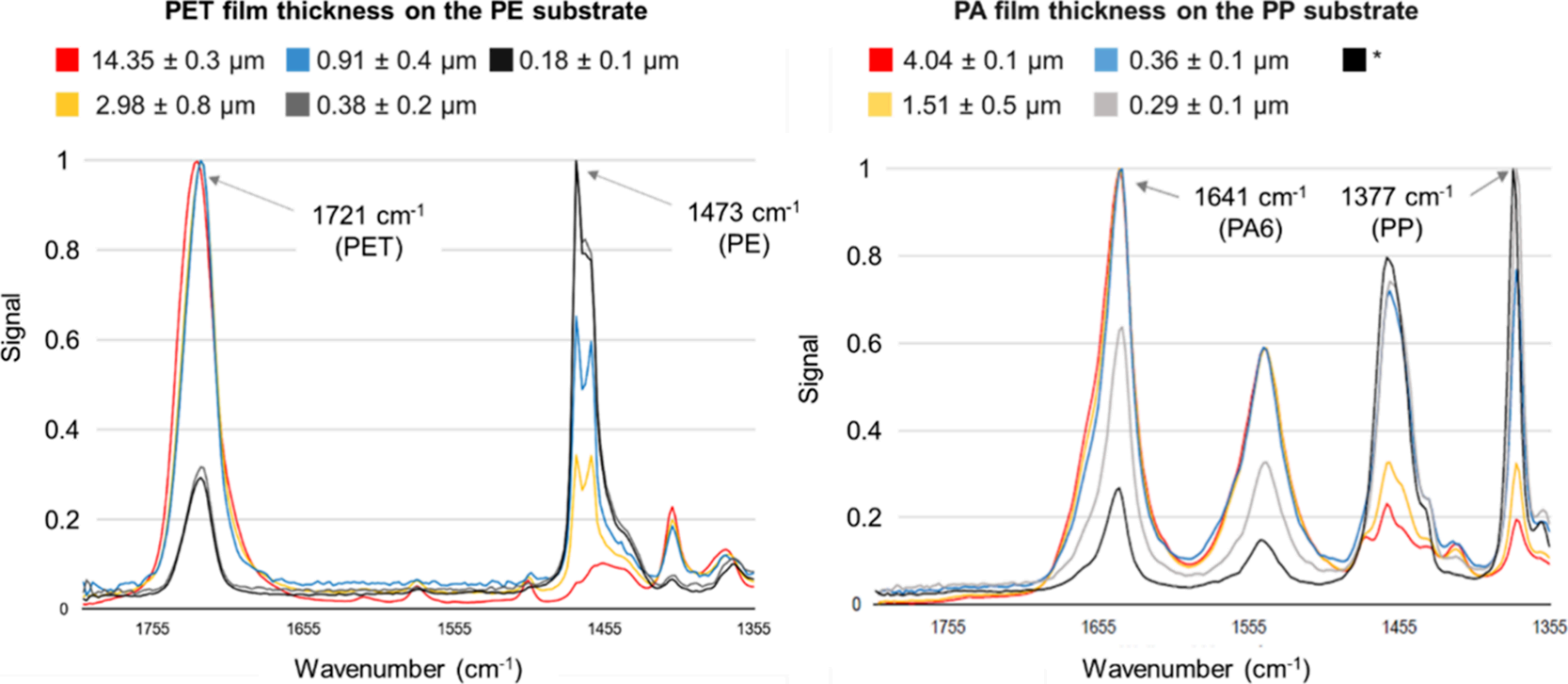
O-PTIR spectra of the PET and PA6 films on the PE and PP substrates dependent on the plastic concentration of the solutions. *The thickness could not be measured.

In Figure 6, bars show the percentage ratios of the normalized peak height of PA6 or PET films to the normalized peak heights of PE and PP, as well as the corresponding absolute normalized peak heights. The peaks are analogous to Figure 2 (i.e., PP – 1377 cm^–1^, PA – 1641 cm^–1^, PE – 1473 cm^–1^, PET – 1721 cm^–1^). Since the calculation is purely mathematical, the O-PTIR signals of PA6 and PET at the wave numbers characteristic of PE (1473 cm^–1^) and PP (1377 cm^–1^) are not on the baseline and are therefore included in the calculation so as not to distort the calculation results. Similarly, this is observed in the case of pure substrates.

**Figure 6:**
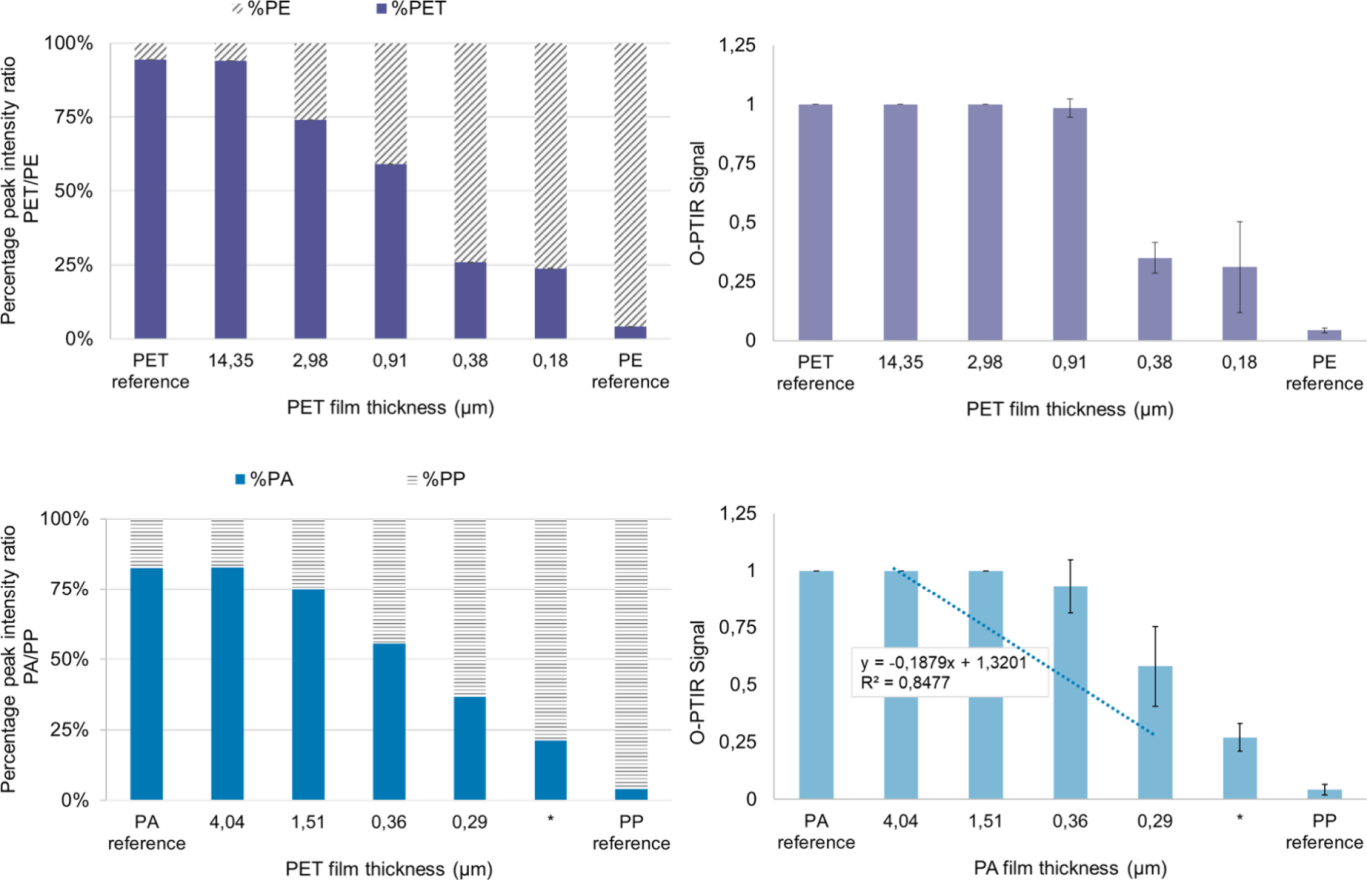
O-PTIR peak ratio and normalized peak intensity of the plastic film as a function of film thickness. *The thickness could not be measured.

In the PA/PP combination, the ratio of the peak intensities of the PA and PP signals shows an almost linear correlation with the measured PA layer thickness, which is reflected in a coefficient of determination of *R* ^2^ = 0.8477, Figure 6. This observation confirms that the chosen methodological approach is suitable for measurements on PA and that the minimum thickness for the detection of O-PTIR on PA is clearly below 0.29 μm. This is because 0.29 μm is the lowest PA film thickness from the solution with 0.125 wt % of PA that could be examined with SEM, and the film thickness from the solution with 0.0625 wt % could no longer be measured with SEM but did provide an O-PTIR signal.

The result with PET/PE combinations is like PA/PP, and the peak intensity decreases overall with decreasing film thickness, but not as smoothly as with PE/PE. For example, the PET peak intensity difference of films produced from 1 to 0.5 wt % is significantly lower than the film thickness difference, i.e., a PET film with a layer thickness of approximately 14 μm and approximately 3 μm produces a similarly high O-PTIR signal. For the approximately 14 μm thick film (1 wt % PET), no signal from the substrate is detected. In the case of the thinnest film thickness on PE of approximately 0.18 μm, there is still a clearly recognizable characteristic signal for PET.

Similar results have been described in the literature for other polymer films of varying thickness measured with different IR technologies in reflection mode.^16,28^ In particular, the change in the reflection–absorption IR signal is not perfectly proportional to the change in film thickness and depends, among other things, on the thickness range. For example, if the thickness of PS increases to approximately 1 μm, the IR signal growth begins to decline. This means that the film approaches or exceeds a certain fraction of an effective “optical depth” or when the film is so thick that further IR absorption does not contribute significantly to the observed IR reflection–absorption signal.^28^ In the case of the infrared frustrated total internal reflection studied on PS films in the range of 0.1–5 μm, it has been shown that this type of IR signal is roughly proportional in the thin limit, i.e., for very thin layers (approaching ∼0.1 μm), the absorption band intensities scale approximately with thickness.^16^ That is, doubling the thickness gives approximately double the signal for that thin range, which is consistent with a low-absorbance regime where attenuation of the IR beam is small. At the same time, as the thickness becomes larger (near the upper limit of 5 μm), the signal does not increase linearly anymore, and the growth in band intensity with further increases in film thickness is not proportional. According to the authors, this occurs because as the film thickens, a larger portion of the evanescent field is absorbed or frustrated over (i.e., within) the film, so that additional material contributes less to additional absorption beyond a certain point.^16^ Overall, the literature reports that for thin films <∼1 μm, the relationship between the thickness and the IR signal is close to linear. Above ∼1–2 μm, increasing thickness yields diminishing incremental signal, i.e., each additional μm adds less than the previous μm, and the upper end (5 μm) still shows further increase, but much less than linear extrapolation of the low-thickness slope would predict.^16^ Overall, the O-PTIR measurements correspond very well with these findings, as the heights of the characteristic peaks of the films produced from the solutions with 0.5% and 1% by weight (film thickness ≥2.98 μm for PET and ≥1.51 μm for PA6) do not differ significantly. The average height of the peaks of thinner layers shows an almost linear behavior across the varied concentration.

Considering the film area (Figure 3) and film thickness (Table 2), it is evident that PET films have a larger area and greater film height than PA6 films, even though they are produced from solutions with the same concentrations. Since the density of PET is 1.34 g/cm^3^ and the density of PA6 is 1.14 g/cm^3^, a reverse trend would be expected at first glance. Primarily, since the film thickness is determined only at the points where the O-PTIR measurements are carried out, i.e., in the middle of the film, these results do not represent the overall picture. Consequently, it may be that the overall average film thickness and area (or volume) of the entire films do correspond to the expected trend after all. The varying layer thickness can be a consequence of the reasons already mentioned, which affect film formation and morphology, as well as shrinkage during drying, crystallization, molecular weight and viscosity differences, etc.^25–27^ Consequently, this aspect should be analyzed in more detail in a separate work.

## 6 Conclusions

The aim of this feasibility study is to determine the dependence of the O-PTIR signals on film thickness for the commonly used plastics PA6 and PET. Drops with different concentrations of these plastics in HFIP are applied separately to two substrates, PP and PE, using the drop deposition method. The expectation is that as the film thickness decreased, the O-PTIR signal of the film would also decrease and the signal of the substrate would increase until no signal of the film can be recognized. This expectation is only partially confirmed. The overall aim of the study, to determine the minimum detectable film thickness, could not be achieved because the sample preparation approach used reached its limits. In particular, this means that it has not been possible to produce sufficiently thin and stable films using the drop separation method to achieve the detection limit of the O-PTIR.

The main results of this study show that the thinnest layer thicknesses that could be produced in this work, approximately 0.18 μm PET and <0.29 μm PA6, exhibit clearly recognizable O-PTIR signals. At the same time, it is important to note that this study is, first of all, a feasibility study with the aim of verifying technical implementation.

Based on these findings, the sample preparation method should be optimized in subsequent works. This can be achieved by other film manufacturing methods or optimizing the drop deposition method used, e.g., by determining more optimal environmental conditions or using other more compatible coating/substrate combinations. In addition to the aspects already mentioned above, the interaction between the substrate and the coating and the influence of this interaction on the resulting O-PTIR signal should also be investigated in detail. Furthermore, smaller variations in film thickness should be investigated in order to increase the reliability of the relationship between the IR absorption peak heights of the coating and substrate, on the one hand, and the thickness, on the other. Finally, the influence of the measurement condition such as IR and probe laser powers on the detectability of the polymer films should be addressed.

## Supplementary Material









## Notes

Raw data from O-PTIR spectra of all tested specimens and SEM images of films (PDF)
Raw data from O-PTIR spectra of PA-PP (XLSX)
Raw data from O-PTIR spectra of PET-PE (XLSX)
Raw data from O-PTIR spectra of PA_PET_PP_PE references (XLSX)
