# Supplementary material for: Influence of Layer Thickness on the Detection of Plastic Films Using Optical Photothermal Infrared Spectroscopy (O-PTIR)
Source: ACS Meas Sci Au. 2026 Jan 22;6(1):150–7. doi: 10.1021/acsmeasuresciau.5c00149 (PMC12921621; doi:10.1021/acsmeasuresciau.5c00149)
Supplement: Supplementary file 1 [file tg5c00149_si_001.pdf]

Suppporting Information

Influence of layer thickness on the detection of plastic films using optical photothermal infrared spectroscopy (O-PTIR)

Jan Fridtjof Häusler[a], Florian Bittner[a], Madina Shamsuyeva[a]*

[a] IKK – Institute of Plastics and Circular Economy of the Leibniz University Hannover, An der Universität 2, 30823 Garbsen

*Corresponding author: shamsuyeva@ikk.uni-hannover.de

TABLE OF CONTENTS

1. SEM-imaging of the tested samples 1
2. Raw data of the O-PTIR measurements 2

1. SEM-imaging of the tested samples

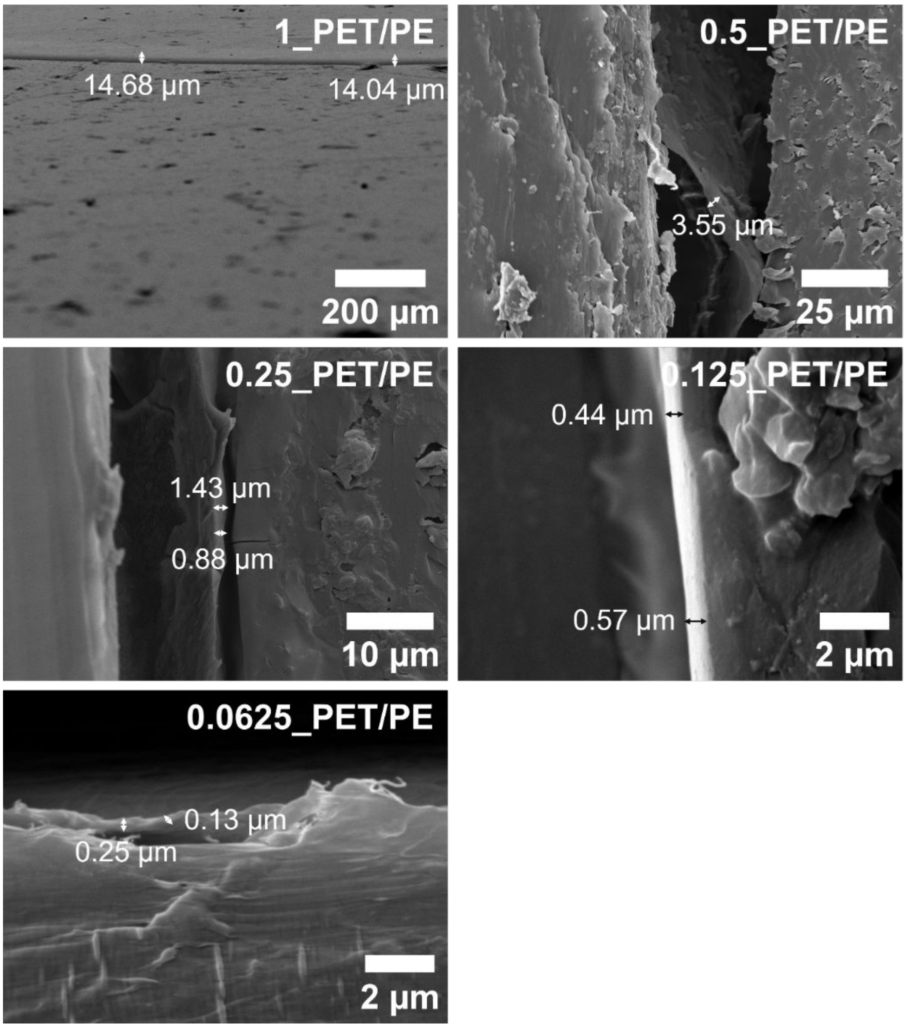

Figure S1: Exemplary SEM images of the PET films

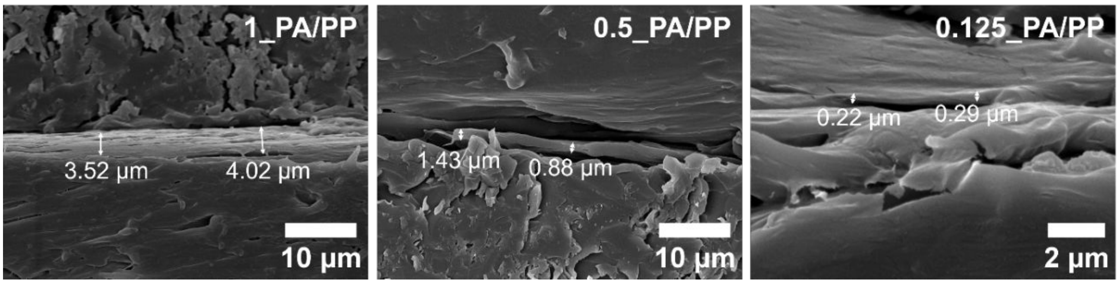

Figure S2: Exemplary SEM images of the PA films

2. Raw data of the O-PTIR measurements

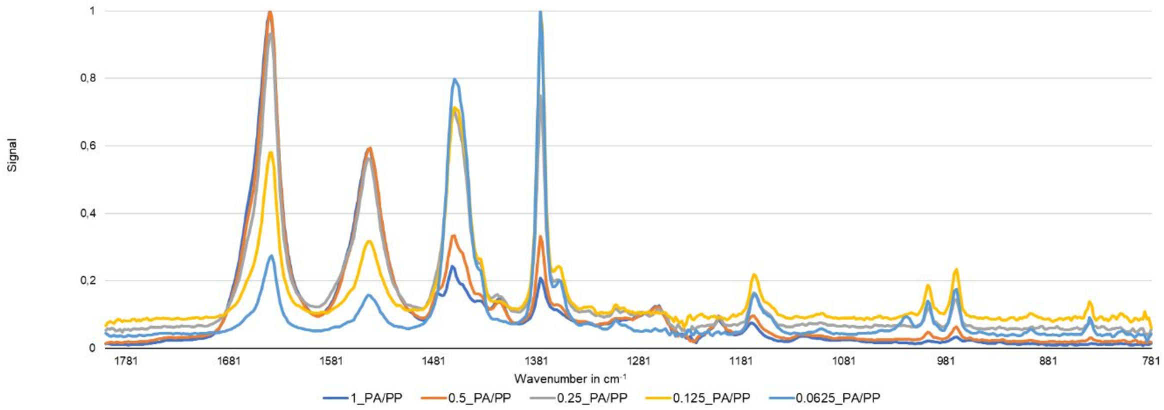

Figure S3: Raw O-PTIR spectra of the PA/PP

Raw data on O-PTIR
measurement of PA-

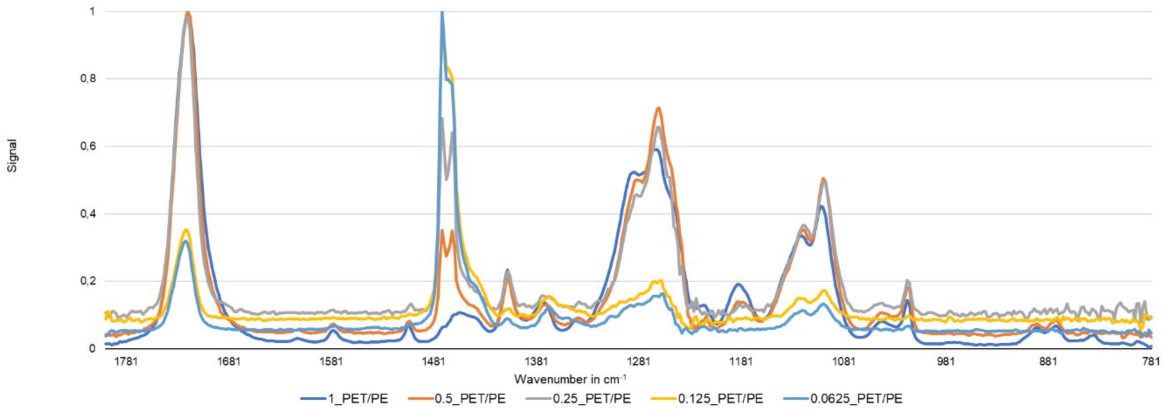

Figure S4: Raw O-PTIR spectra of the PET/PE

Raw data on O-PTIR
measurement of PET

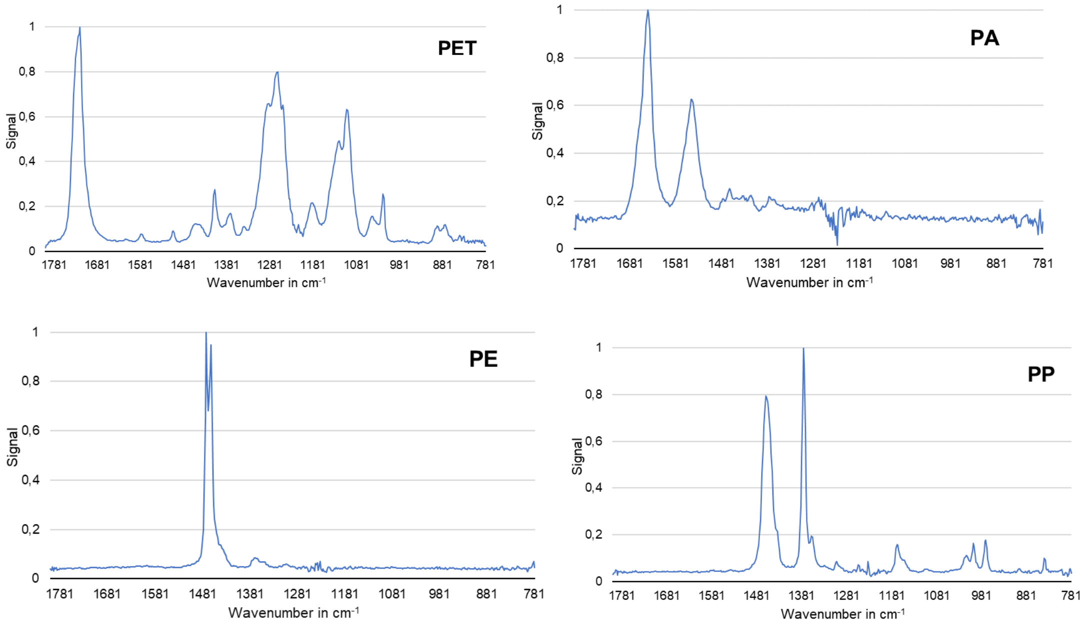

Figure S5: Raw O-PTIR spectra of the references PET, PA, PP and PE

Raw data on O-PTIR
measurements of PA
